# Supplementary material for: Accuracy of Pulse Oximetry in the Presence of Fetal Hemoglobin—A Systematic Review
Source: Children (Basel). 2021 Apr 30;8(5):361. doi: 10.3390/children8050361 (PMC8145233; doi:10.3390/children8050361)
Supplement: Supplementary file 1 [file children-08-00361-s001.zip › children-1151401-supplementary-figure S1.pdf]

((newborn OR neonate OR preterm OR term OR infant) AND (HbF OR hemoglobin F OR fetal hemoglobin) AND ("after birth" OR postnatal) AND (oxygenation OR arterial oxygen saturation OR pulse oximetry OR SaO2 OR SpO2))

Search strategy for PubMed: last performed January 24th, 2021

No limits activated

#1 MeSH descriptor newborn explode all trees

(result: 780 284)

#2 MeSH descriptor neonate explode all trees

(result: 774 174)

#3 MeSH descriptor term OR preterm explode all trees

(result: 1 225 736)

#4 MeSH descriptor infant explode all trees

(result: 1 270 689)

#5 (#1 OR #2 OR #3 OR #4) (result: 2 567 971)

#6 MeSH descriptor HbF explode all trees

(result: 2 605)

#7 MeSH descriptor hemoglobin F explode all trees

(result: 9 955)

#8 MeSH descriptor fetal hemoglobin explode all trees

(result: 9 812)

#9 ((#6 OR #7 OR #8)) (result: 11 110)

#10 MeSH descriptor „after birth“ explode all trees

(result: 41 191)

#11 MeSH descriptor postnatal explode all trees

(result: 123 292)

#12 ((#10 OR #11)) (result: 155 434)

#13MeSH descriptor oxygenation explode all trees

(result: 754 101)

#14 MeSH descriptor arterial oxygen saturation explode all trees

(result: 16 722)

#15 MeSH descriptor pulse oximetry explode all trees

(result: 20 350)

#16 MeSH descriptor SaO2 explode all trees

(result: 4 400)

#17 MeSH descriptor SpO2 explode all trees

(result: 6 608)

#18 (#13 OR #14 OR #15 OR #16 OR # 17) (result: 764 011)

#19 ((#5) AND (#9) AND (#12) AND (#18)) (result: 1566)
